# The Essentials of Sanatorium Treatment

**Published:** 1901-12-28

**Authors:** 


### THE ESSENTIALS OF SANATORIUM TREATMENT.

Some confusion is apt to arise as to what is meant by the term sanatorium treatment as applied to consumption, from the fact that the methods in use at different places are not always the same. More especially is this the case in regard to some of the German institutions where, following the ordinary practice of that country, the tendency has been to distinguish the various methods by the names of their chief exponents, and what is far worse to restrict the treatment attainable at each institution to the particular precepts of the master of that particular cult. This is a serious danger to patients, and a serious embarrassment in the choice of a sanatorium, for it is often by no means easy to find one in regard to which locality, doctor, and method shall all alike be suitable for the case in hand. At the Congress of Tuberculosis, Dr. R. W. Philip, physician to the Victoria Hospital for Consumption, Edinburgh, gave a useful account of the various factors involved all of which have to be considered if sanatorium treatment is to attain its full utility.

*Open Air.*—Whatever may be said to the contrary, or by way of qualification, hyperaëration is the major and essential agent in the treatment, the *conditio sine quâ non.* There may be differences as to diet, rest, and exercise, but the uninterrupted exposure of the patient to the influence of fresh air is applicable in every case. In early days there was some timidity about this. Efforts were made to attain a fairly constant temperature in dwelling and bedrooms, and it was an instruction to nurses that the temperature should be regulated by the thermometer placed in each room. All this, however, has been greatly modified by experience. The freest access of fresh air is to be allowed to the patient day and night, at all seasons, provided he is sufficiently clad. Indeed there seems a disadvantage in endeavouring to combine free exposure out of doors with the occupancy of rooms whose temperature is maintained constant. The patient should be exposed indoors to approximately the same conditions as out of doors. Let him be made comfortable by means of clothing or even hot water bottles, "but let us be sparing of artificial heating of the atmosphere." This side of the problem is summed up by saying "there cannot be too free exposure to the open air."

*Rest and Release from Disturbing Influences.*—For almost all patients rest is the immediate indication when treatment is commenced, and must be maintained as part treatment at least for a long time. The pulse and temperature are the chief guides. The patient should rest chiefly if the temperature oscillate above 100·5° Fahr., or if the pulse rate be maintained above 90 per minute. It is remarkable how soon the visits of friends or the receipt of letters may exercise a definitely harmful influence.

*Exercise.*—As improvement succeeds, as evidenced more particularly by pulse and temperature, the patient is allowed to take short turns, measured at first by minutes, once or twice in the day; at first on a level and gradually on an incline, and even a hill. By degrees the amount of exercise is increased, the effect being gauged by careful record of pulse and temperature.

*Dietary.*—The alimentary disturbances which form so marked a feature of the disease must be corrected by a carefully studied dietary. The patient must be induced to take a sufficient quantity—a very different thing from the "forced feeding" of which one sometimes hears. If physiological conditions are fulfilled the patient generally eats of his own accord. He must rest sufficiently before and after meals, the bowels must be regulated with care, and it is often an advantage for the meals to be taken in the open air.

*Skin Hygiene.*—Attention to the skin is not a mere matter of cleanliness. Skin activity is demanded both for heat regulation and for excretion. The clothing should be both warm and porous. Shetland wool is an excellent example of the sort of thing that is wanted. The absurd over clothing which is sometimes sanctioned is to be condemned. The regular use of a rapid cold bath is generally highly beneficial, but care must be taken that the patient is quickly dried after the bath without over effort to himself.

The results described by Dr. Philip are admirable, and it is a matter of great importance to find that it is by no means necessary, however desirable it may seem in some cases, that sanatoria should be in distant country places, or in mountain districts. The two sanatoria to which Dr. Philip more especially refers in his article are within three miles of the centre of Edinburgh.

